# Amazonian chemical weathering rate derived from stony meteorite finds at Meridiani Planum on Mars

**DOI:** 10.1038/ncomms13459

**Published:** 2016-11-11

**Authors:** Christian Schröder, Phil A. Bland, Matthew P. Golombek, James W. Ashley, Nicholas H. Warner, John A. Grant

**Affiliations:** 1 Biological and Environmental Sciences, Faculty of Natural Sciences, University of Stirling, Stirling FK9 4LA, UK; 2 Department of Applied Geology, Curtin University, Perth, Western Australia 6845, Australia; 3 Jet Propulsion Laboratory, California Institute of Technology, Pasadena, California 91109, USA; 4 Department of Geological Sciences, State University of New York at Geneseo, Geneseo, New York 14454, USA; 5 Center for Earth and Planetary Studies, National Air and Space Museum, Smithsonian Institution, Washington, District Of Columbia 20560, USA

## Abstract

Spacecraft exploring Mars such as the Mars Exploration Rovers Spirit and Opportunity, as well as the Mars Science Laboratory or Curiosity rover, have accumulated evidence for wet and habitable conditions on early Mars more than 3 billion years ago. Current conditions, by contrast, are cold, extremely arid and seemingly inhospitable. To evaluate exactly how dry today's environment is, it is important to understand the ongoing current weathering processes. Here we present chemical weathering rates determined for Mars. We use the oxidation of iron in stony meteorites investigated by the Mars Exploration Rover Opportunity at Meridiani Planum. Their maximum exposure age is constrained by the formation of Victoria crater and their minimum age by erosion of the meteorites. The chemical weathering rates thus derived are ∼1 to 4 orders of magnitude slower than that of similar meteorites found in Antarctica where the slowest rates are observed on Earth.

## Abstract (web summary)

Little is known about the impacts of Mars' contemporary dryness on weathering processes. Here, using iron oxidation estimates from the Mars Rover Opportunity, the authors quantify chemical weathering rates for Mars, finding appreciably slower rates compared with the lowest values on Earth.

## Graphical abstract

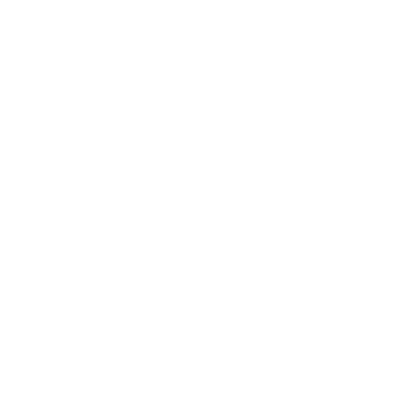

Recent mineralogical and geochemical investigation of the Martian surface have confirmed the view that ancient Mars was much more Earth-like, that is, had abundant liquid water at its surface and provided generally habitable conditions^1^,^2^,^3^,^4^,^5^. The question then arises whether life began on Mars as well as Earth, and whether there could still be extant life despite the currently prevailing extremely arid conditions. Establishing a current chemical weathering rate for rocks as a measure of available water moisture is thus important. Although many Martian rocks exhibit signs of chemical weathering, this is difficult because it is generally not known when and for how long they experienced a certain set of weathering conditions, which also would have changed over long periods of time.

Mars' atmosphere is sufficiently dense to expect finding meteorites on its surface^6^, and both Mars Exploration Rovers (MERs), Spirit and Opportunity, as well as the Mars Science Laboratory or Curiosity rover have discovered meteorites^7^,^8^,^9^,^10^,^11^. Most of these are iron meteorites but Opportunity also came across several stony meteorite fragments^7^,^8^,^9^. Meteorite finds of a known type—whose unweathered composition is well known from the study of other specimens of the same type on Earth—provide standard samples that have been exposed to the weathering environment of the surface area they fell on^12^,^13^,^14^. Most common meteorite types, for example, ordinary chondrites, contain iron only in its Fe(0) and Fe(II) oxidation states^15^, so that any Fe(III) can be taken as a measure of chemical weathering. Furthermore, because metallic iron oxidizes quickly in the presence of water, they provide a sensitive tracer for the presence of liquid water^16^. On Earth, terrestrial ages of meteorites can be obtained from cosmogenic radionuclides and, combined with the degree of alteration, a chemical weathering rate can be determined.

Here we date the surface exposure ages of meteorites from Mars. The stony meteorites discovered by Opportunity are probably paired^8^ and therefore fell at the same time. Because of their distribution surrounding Victoria Crater, they may be part of the impactor that created that crater. Its age thus defines the meteorites' maximum exposure age. Their degree of physical weathering provides a minimum exposure age. We apply this exposure age bracket and compare their Fe(III) content to the Fe(III) content in meteorites with known exposure age recovered from different terrains on Earth. The chemical weathering rates thus derived on Mars are ∼1 to 4 orders of magnitude slower than the slowest rates in any environment on Earth.

## Results

### Determination of exposure age

**Figure 1:**
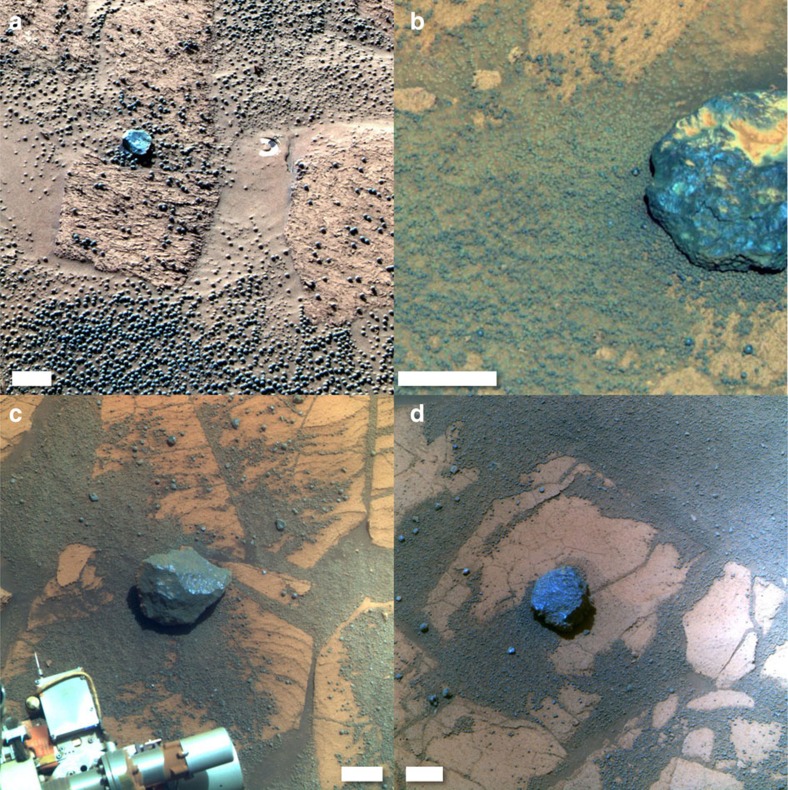
Stony meteorites at Meridiani Planum. False colour Pancam images of the candidate stony meteorites (**a**) Barberton (acquired on sol 123 with sequence P2535), scale bar is 5 cm; (**b**) Santa Catarina (sol 1,055, sequence P2564), scale bar is 6 cm; (**c**) Santorini (sol 1,748, sequence P2597), scale bar is 4 cm; and (**d**) Kasos (sol 1,884, sequence P2574), scale bar is 4 cm (credit: NASA/JPL/Cornell).

The stony meteorites (Fig. 1) were identified on the basis of their metallic iron content. Although they were discovered serendipitously dispersed across almost 10 km, their chemical and mineralogical composition is virtually identical^7^,^8^,^9^. Their chemistry is most similar to the HED group of meteorites albeit with an elevated (Fe, Ni) metal content^7^,^8^. They thus resemble the silicate component of mesosiderites, which are a group of stony-iron meteorites^7^.

**Figure 2:**
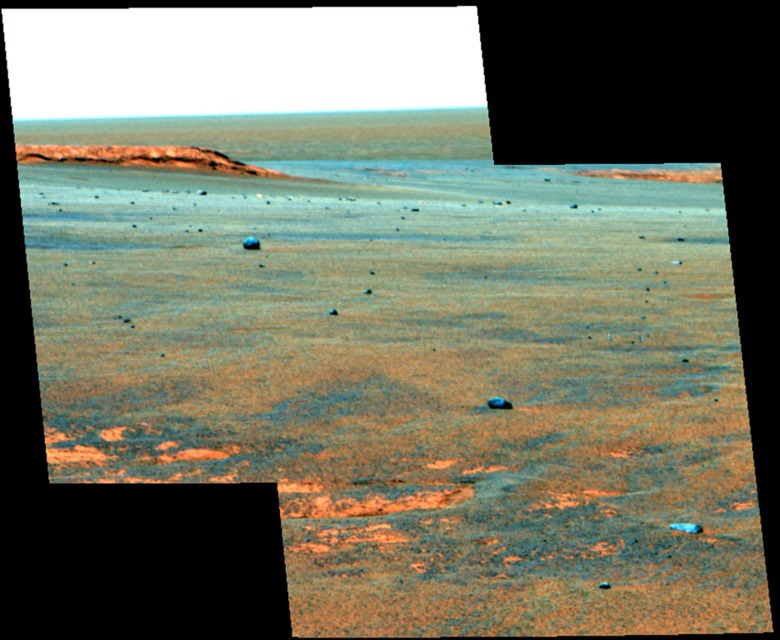
Meteorite accumulation at Victoria Crater. False colour, seam corrected Pancam mosaic showing the Santa Catarina cobble field at the rim of Victoria crater, which is visible on the right hand edge of the mosaic. The images were acquired on sol 1,049 with sequence IDs P2555 and P2556 (credit: NASA/JPL/Cornell).

The four meteorites are found on a diversity of terrains that make up the area traversed by the Opportunity rover^17^. Santa Catarina and similar cobbles^7^,^18^ appear strewn across the smooth, sandy annulus around the Victoria impact crater (Fig. 2). Barberton is found on the eroded rim of Endurance crater on smooth terrain with small ripples about 6.2 km north of Victoria crater. Santorini and Kasos are ∼1.5 and ∼3.3 km south of Victoria crater, respectively, in large rippled terrain. Because the meteorites appear paired based on their chemistry^8^, they may be part of the same fall. One possibility is that they are fragments of the impactor that formed Victoria crater as suggested by their similar distribution to fragments around terrestrial impact craters^8^,^19^. This may be difficult to reconcile, however, with their occurrence on top of the annulus at Victoria that was produced by erosion^20^. An alternate possibility is that their arrival times post date the age of the terrains on which they are found, which is suggested by their occurrence on all the different sand and outcrop surfaces, and their relatively unweathered appearance compared with iron meteorites found in the same region^10^. Barberton likely arrived at Endurance after the terrains formed and after the crater was eroded into its present form^21^. The meteorite is on the rim of Endurance crater whose overturned flap and ejecta have been eroded and covered with sand to make the smooth surface with small ripples. Furthermore, Endurance has experienced considerably less total erosion than Victoria^20^,^21^ and is therefore younger given their proximity and similarity in target materials. Moreover, Barberton is not on a pedestal like the iron meteorite Block Island^10^ and adjacent surfaces are not significantly scoured as might be expected given the ease with which surrounding rocks are eroded^17^,^20^. Nevertheless, these two hypotheses constrain other intermediate possibilities (that is, the meteorites fell after some of the Meridiani terrains formed), and we will consider both hypotheses to constrain the maximum and minimum meteorite exposure age on the surface.

If the meteorites are fragments of the impactor that formed Victoria crater, the age of the crater would be their surface exposure age. Given that Victoria crater impacted into Burns formation sulfate sandstones^20^, which form the light-toned bedrock of Meridiani Planum, the crater retention age of the bedrock (∼70 Myr; ref. 17) would provide an upper limit. The smooth sandy surface of the Victoria crater annulus formed after ejected blocks of sulfate sandstone were eroded down about 1 m until granule-sized blueberry concretions that had weathered out of the bedrock inhibited further erosion^20^. Using the same methods for determining the crater age of other Meridiani terrains^17^, we find a 40 Myr model age for the smooth inner annulus around Victoria. We estimate the annulus took about 10 Myr to form by applying the eolian erosion rate for Meridiani over the past 10 Myr (0.1 m per Myr)^17^ to erode 1 m of sulfate sandstone. This yields an impact age of around 50 Myr ago for Victoria crater, which is less than the maximum age from the age of the bedrock (70 Myr ago), as would be expected given the moderately modified form of the crater^20^.

**Figure 3:**
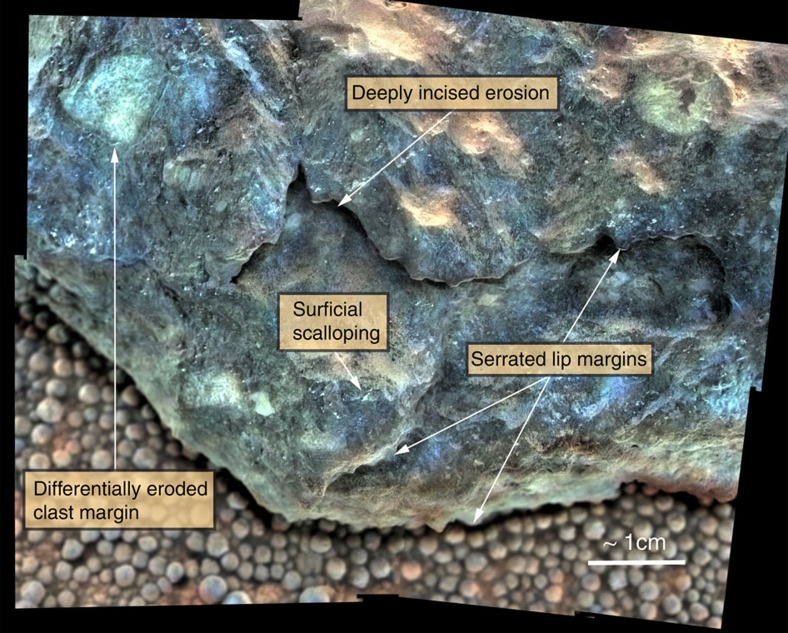
Physical weathering of Santa Catarina. Microscopic Imager mosaic of Santa Catarina merged with Pancam colour images (acquired on Sol 1,055 with sequence p2564). Visible are signs of deep incision, differential erosion, and scalloping, producing serration along rock margins. Note the absence of any residual fusion crust or obvious regmaglypts. Note also the highly brecciated structure of the rock. Figure modified from ref. 8.

If the meteorites fell after the terrains on which they are found had formed, we can use the age of the terrains as their maximum exposure age. The smooth terrain is about 23 Myr old and the large ripple terrain is about 18 Myr old (ref. 17), so the maximum exposure age is around 20 Myr. If the meteorites fell well after the terrain age of 20 Myr, it would be helpful to estimate the minimum exposure age. We use two methods to constrain the minimum exposure age. First, because Barberton likely postdates the erosion of Endurance crater into its current form, the inferred age of 2–4 Myr for the crater based on its morphology and crater counts^17^ sets an upper limit to its exposure age. Second, stereo MI images indicate that of order 1 cm of the lowermost portion of Santa Catarina has been eroded away (Fig. 3). These surface modifications could have occurred as the result of eolian abrasion alone or as a combination of abrasion and aqueous alteration as indicated by the ferric iron. They demonstrate that the rock probably had an extended residence time on the Martian surface. Figure 3 also shows the highly brecciated structure of the rock, which likely contributes to differential mass removal by providing strength variations throughout the rock volume. The rate of abrasion to form ventifacts in hard rocks at the Pathfinder landing site was estimated to be on the order of 10^−4^–10^−5^ m per Myr (ref. 22). Because the rate of abrasion at a site is dependent on both the sand supply and the frequency of winds strong enough to saltate sand, we adjust the rate of abrasion by the difference between the long-term erosion rates at Mars Pathfinder and Meridiani (about 2 orders of magnitude), which is dependent on the same two factors^20^,^22^. This would suggest abrasion rates of hard rocks at Meridiani of order 10^−2^–10^−3^ m per Myr, which would correspond to 1–10 Myr to erode the base of Santa Catarina. As a result, the 2–4 Myr and 1–10 Myr ages for Barberton and Santa Catarina, respectively, would represent the minimum exposure age for the stony meteorites on Mars.

### Chemical weathering rate and comparison to Earth

We used the ferric iron content (Table 1; as published by Schröder and co-workers^8^) obtained with Opportunity's Mössbauer spectrometer^23^ in these meteorites (see Methods) as a measure of alteration since their fall. It is possible that a fraction of this Fe(III) stems from the formation of a fusion crust^24^. However, it is unclear whether passage through Mars's CO_2_ atmosphere would have led to the formation of an oxidized fusion crust at all, and there is no obvious evidence for a fusion crust^8^ or regmaglypts (Fig. 3). Some Fe(III) might have been removed by wind abrasion, on the other hand, a process suggested to have affected Santa Catarina and the iron meteorites at Meridiani^10^. We take the average amount of Fe(III) measured in the stony meteorite fragments and derive a chemical weathering rate of 9±4% Fe(III) formation per 50 Myr or per 1–20 Myr if they are either part of the impactor that created Victoria crater or if they fell after the terrains on which they are found had formed, respectively. That translates into a rate of 0.18% per Myr Fe(III) formation (for 50 Myr) to 9% per Myr (for 1 Myr) with an average of 0.45% per Myr (for 20 Myr) if a linear rate is assumed. Note, however, that studies of weathering in meteorites in terrestrial settings (see below) show that Fe oxidation does not progress in a linear fashion where rapid initial oxidation is followed by passivation^12^,^13^,^14^.

**Figure 4:**
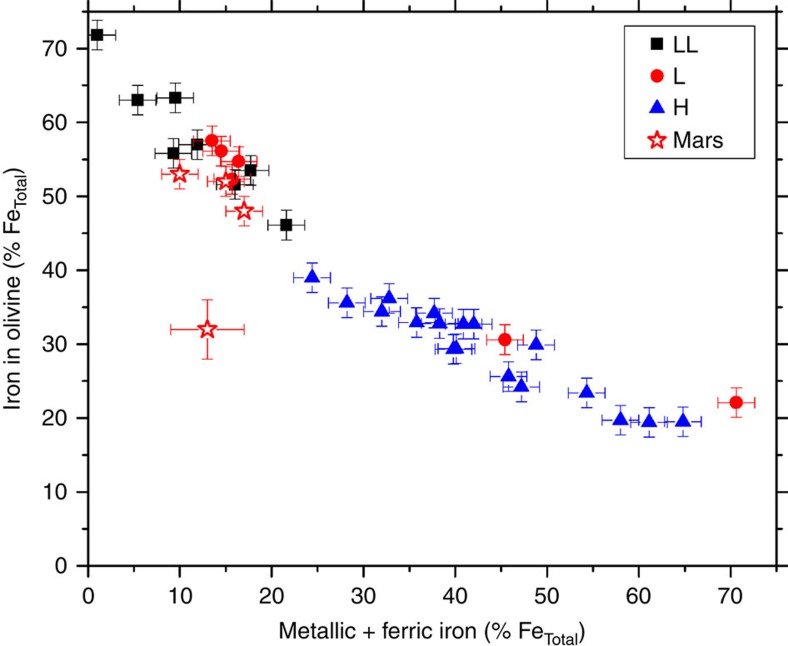
Distribution of Fe in ordinary chondrites and stony meteorite finds on Mars. The plot shows the amount of ferrous iron in olivine (percentage of total iron) as a function of metallic iron plus ferric iron (percentage of total iron) in ordinary chondrites returned from the Larkman Nunatak region in Antarctica (filled black squares: LL chondrites; filled red circles: L chondrites; filled blue triangles: H chondrites) and compares it to the stony meteorite finds on Mars (open red stars). The error bars represent the general uncertainty of ±2% absolute except for the data point representing Kasos where the uncertainty is ±4% (compare Table 1).

To put this number into perspective we need to compare it to studies of similar groups of meteorites collected from various weathering environments on Earth. Bland and co-workers have done several such studies using ordinary chondrites^12^,^13^,^14^. Ordinary chondrites are divided into three geochemically and mineralogically distinct groups: H chondrites have a high Fe content with a high metallic Fe versus FeO in silicates ratio; L and LL chondrites have a lower Fe content with less Fe metal and more FeO in silicates. The stony meteorites at Meridiani are not ordinary chondrites but when we compare them to a Mössbauer spectroscopy survey of chondrites^25^ (see Methods), the distribution of iron between mineral phases is the same as that of L and LL chondrites (Fig. 4). In Fig. 4, L and LL chondrites are nicely separated from H chondrites. Two L chondrites plot within the field of H chondrites, which is most likely a result of more severe chemical weathering that not only oxidized metallic iron but also some ferrous iron in olivine. The stony meteorites discovered at Meridiani plot within the field of the L and LL chondrites. One of these stony meteorites, Kasos, has less iron in olivine and instead more iron in pyroxene than the others (Table 1). Because there is no difference in chemical composition, this may simply reflect some heterogeneity in mineral grain distribution on the scale of the field of view of the Mössbauer instrument (∼1.4 cm).

**Figure 5:**
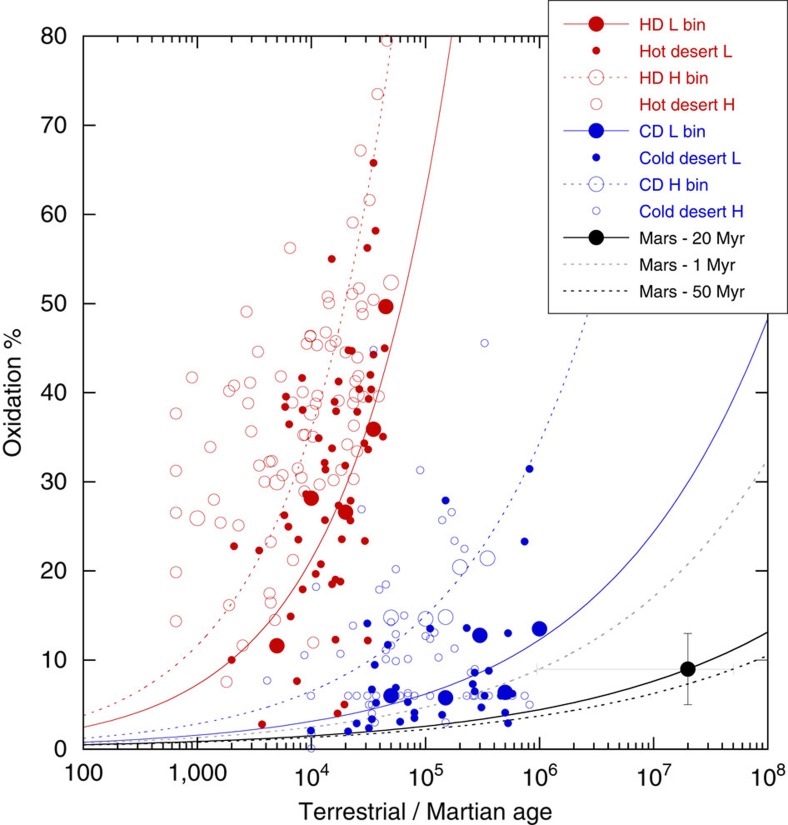
Fe(III) as a function of exposure age. This plot shows the abundance of ferric iron (Oxidation) in several groups of meteorites as a function of their exposure age on the surface of Earth^12^,^13^,^14^ or Mars. Filled red and blue circles are L chondrites, open red and blue circles are H chondrites, the filled black circle is the average of the stony meteorites found on Mars. Red denotes meteorites recovered from hot deserts (HD), blue meteorites recovered from cold deserts (CD). Large red and blue circles are age-binned averages, small red and blue circles data from individual meteorites. The solid and dotted red and blue lines are trend lines fitted to the age-binned L chondrite and H chondrite data points, respectively. The black solid line is a trend line fitted through the Martian data point at an age of 20 Myr whereby the grey and black dotted lines are trend lines for the minimum and maximum exposure ages, respectively. The error bars reflect the uncertainty in the Fe(III) content (±4%, compare Table 1) and stretch to the minimum (1 Myr) and maximum (50 Myr) exposure ages.

In Fig. 5 we plot the fraction of ferric iron formed as a function of time for H chondrites and L chondrites investigated by Bland and co-workers^12^,^13^,^14^, and the stony meteorites at Meridiani. Because ferric iron was determined from surface measurements in the Meridiani meteorites and from subsurface measurements in the terrestrial finds (see Methods), and because weathering proceeds along surfaces and follows fractures into a rock, the MER measurements may represent an overestimation compared with the terrestrial measurements. We distinguish between meteorites recovered from hot desert regions on Earth (Sahara, Australia, SW, USA) and cold desert regions (Antarctica) in Fig. 5. Both areas represent slow terrestrial chemical weathering rates. The plot shows that H chondrites weather faster in all environments than L chondrites because they contain more metallic iron (for example, Fig. 4) than L chondrites. Metallic iron is the most sensitive phase towards chemical weathering. Weathering rates in Antarctica are significantly slower than those in hot desert regions on Earth. The Antarctic rate arguably represents the slowest chemical weathering rate anywhere on Earth. If we apply the shortest possible residence time (1 Myr) for the stony meteorites at Meridiani and compare at 9% oxidation, the Martian rate approaches the Antarctic rate. The Martian rate becomes significantly slower the longer the true residence time is. If we apply the time of formation of Victoria Crater (50 Myr ago), the Martian rate would be two orders of magnitude slower than the Antarctic rate. The weathering rates diverge as oxidation progresses with time. At 50% oxidation, a level where ordinary chondrites start to disintegrate (see below), Martian weathering rates could be up to 4 orders of magnitude slower than the Antarctic rate.

## Discussion

Average long-term physical erosion rates on Mars since the Noachian are 2–3 orders of magnitude slower than the slowest erosion rates in any environment on Earth (minimum ∼1 m per Myr) and are consistent with the present day dry and desiccating environment^17^,^26^. Erosion over the past 20 Myr at Meridiani Planum is dominated by eolian activity and abrasion with no evidence for liquid water^17^,^26^, consistent with the extremely slow chemical weathering of iron.

This bodes well for the preservation of Mars' ancient rock record throughout the Amazonian. Bland and Smith^6^ had predicted that meteorites on Mars might survive on the order of several billion years. Figure 5—where we chose a residence time of 20 Myr with an error bar stretching out to the minimum and maximum residence times of 1 and 50 Myr, respectively—corroborates that prediction. Ordinary chondrites start to disintegrate when the ferric iron content—mostly in the form of iron oxides, that is, rust—reaches ∼50%. They subsequently disappear as recognizable pieces from the rock record. Ordinary chondrites reach that number in hot desert regions on Earth after several 10,000 years. In Antarctica, they may survive for 10–100 Myr if blue ice stranding surfaces were stable over these timescales. On Mars, ordinary chondrites could indeed survive several billion years.

How did the iron in the stony meteorites get oxidized? Mars may currently be in an interglacial period characterized by desiccation of the lower latitudes^27^. Nevertheless, the Mars Science Laboratory (MSL) a.k.a. the Curiosity rover observed seasonal and diurnal moisture exchange between the soil and the atmosphere at Gale crater^28^. Gale is at latitude 4.5° S, almost at the same latitude as Meridiani Planum (2° S). Relative humidity is greatest close to the soil surface, where adsorption and salt hydration likely play a role in moisture exchange^28^. In fact, the observations support the formation of brines in the top 5 cm of soil and are consistent with changes in hydration states of salts in the top 15 cm (ref. 29). The widespread abundance of perchlorates suggest this is common elsewhere on Mars^29^, and the MSL moisture measurements thus reinforce the acid fog model^30^ and suggestions that weathering is enhanced in the shallow subsurface^31^. They are consistent with observations with the Spirit rover in Gusev crater (15° S). Spirit eventually became embedded in a small sand-filled crater after breaking though a soil crust whose formation is consistent with dissolution and precipitation by soil water in the shallow subsurface^32^. Hurowitz and co-workers^33^ argue that a low-pH, low water to rock ratio process leads to leached, yet not chemically fractionated, alteration zones on rock surfaces and explains the soil geochemistry in Gusev crater and elsewhere on Mars. Haskin and co-workers^34^ present evidence for limited interaction of water with the volcanic rocks covering the Gusev crater plains. This evidence includes coatings on one rock, which are enriched in chlorine and ferric iron, and positive correlations between magnesium, sulfur and other salt components. The coatings might have formed through interactions with transient thin films of liquid water, possibly during a period when the rock was buried^31^, and the evidence seems consistent with the transient presence of perchlorate brines and hydration of salts, as suggested for Gale crater^29^.

Water may not be necessary to explain the oxidation of iron, however. In the absence of coatings and where weathering rinds appear to be largely isochemical compared with the underlying unaltered rock, diffusive oxidation may have been driven instead by the oxidation gradient between fresh basaltic rock and an oxidizing atmosphere. This process has been documented in the McMurdo Dry Valleys of Antarctica on Earth^35^. While the oxygen fugacity of the Martian atmosphere is considerably lower than that of the oxygen-rich Earth atmosphere, the oxygen fugacity during the genesis of Martian basalts is also significantly lower compared with Earth. A similar oxidation gradient therefore exists between fresh basalt and atmosphere on Mars.

One or a combination of the processes described above likely acted on the meteorites at Meridiani and is responsible for the oxidation of iron. The significance is that in the meteorites' case we can constrain their duration. The observed level of Fe(III) formation (our proxy chemical weathering rate) of 9±4% Fe(III) formation per 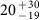 Myr is likely at the lower end for equatorial regions and elsewhere on Mars. There is evidence that some of the iron meteorites at Meridiani experienced episodes of burial by granule ripples^10^, which might have led to the formation of patches of a thin coating^7^,^10^ that appears to be enriched in ferric oxide^36^, or more pronounced chemical weathering that eventually led to cavernous weathering. There is no evidence that the stony meteorites were ever buried though we cannot exclude the possibility. Furthermore, the stony meteorites sit on bedrock or a thin sheet of sand (Figs 1 and 2). The atmosphere-soil water cycle is enhanced within sandy soil^29^. In general, moisture content of atmosphere and soil is increasing with higher latitudes.

Martin-Torres and co-workers^29^ worry that the corrosive effects of chlorine brines might pose a challenge to spacecraft. The extremely slow weathering of meteorites, which contain metallic iron as a phase very sensitive towards chemical alteration, suggests that this is not a threat over the lifetime of a spacecraft, however. The Opportunity rover is testament to that, showing no signs of chemical weathering or corrosion after more than 12 years of operating on Mars (April 2016). The top 5–15 cm of soil should probably be avoided, though, when looking for preserved traces of organic and other volatile compounds.

The chemical weathering rates presented here represent an average over the past 1–50 Myr. Mars may currently be experiencing an interglacial period and probably the driest conditions in equatorial regions^27^. But over the last 10 Myr this may have been preceded by several glacial intervals and a change from a low mean obliquity to a high mean obliquity period^37^,^38^. For the majority of the time there may therefore have been more moisture at the equatorial latitudes of Meridiani than at present. Nevertheless, our chemical weathering rates are ∼1 to 4 orders of magnitude slower than the slowest rates on Earth. Such extreme aridity leads to a drop in the abundance of microbial life to below detection levels even on Earth, as documented for example in the Atacama desert in Chile^39^.

## Methods

### Mössbauer spectroscopy of Martian finds

The MER Mössbauer spectrometers are set up in backscattering geometry and measure the surface of a rock with a circular footprint of 1.4 cm diameter and a sampling depth of 50–200 μm (ref. 23). One spot on each meteorite was investigated with Mössbauer spectroscopy, and the spots were selected so that they fill the field of view of the instrument, are relatively flat, and relatively free of dust^7^,^8^. Fusion crust was not observed on these meteorites. A thin layer of dust does not affect measured Fe(III) concentrations. Mössbauer investigations of the basaltic rocks Adirondack and Humphrey with MER Spirit in Gusev crater in an undisturbed (that is, covered by a thin layer of dust), brushed (dust removed) and abraded condition showed that removing the dust layer did not make a significant difference to the Fe(III) content^40^.

### Iron mineralogy of ordinary chondrites

In a study investigating non-destructive classification approaches for equilibrated ordinary chondrites^25^, 31 out of four groups of ten ordinary chondrites returned from the Larkman Nunatak region in Antarctica (LAR 06470-479; 06500-509; 06570-579; 06820-829) were investigated with a laboratory copy of the MER Mössbauer spectrometer. The meteorites were small hand specimens on the order of several cm in size. They had patches of fusion crust but surfaces free of fusion crust were selected for the analyses. It was assumed that all ferric iron was a result of oxidation of the originally metallic iron. Many of the meteorites exhibited visible rust formation.

### Mössbauer spectroscopy of terrestrial chondrite finds

Bland and co-workers^12^,^13^,^14^ used a transmission Mössbauer spectrometer to analyse powder aliquots obtained from the outer portion of meteorites but below any fusion crust. Powder aliquots were made from 0.2 to 0.5 g of material taken from at least 2 mm below the meteorite surface. Weathering variation with depth was negligible in the small hand specimens used.

### Data availability

The data that support the findings of this study are available from the authors upon request.

## Additional information

**How to cite this article:** Schröder, C. *et al*. Amazonian chemical weathering rate derived from stony meteorite finds at Meridiani Planum on Mars. *Nat. Commun.* **7,** 13459 doi: 10.1038/ncomms13459 (2016).

**Publisher's note:** Springer Nature remains neutral with regard to jurisdictional claims in published maps and institutional affiliations.

## Figures and Tables

**Table 1:** Distribution of iron between mineral phases and oxidation states* †.

**Rock**	**Olivine, Fe**^**2+**^ **(%)**	**Pyroxene, Fe**^**2+**^ **(%)**	**npOx, Fe**^**3+**^ **(%)**	**Kamacite, Fe**^**0**^ **(%)**	**Troilite, Fe**^**2+**^ **(%)**
Barberton	48	32	6	11	3
Santa Catarina	52	26	14	1	6
Santorini	53	28	8	2	9
Kasos	32	41	8	5	14
Average:	46±10†	32±7	9±4	5±5	8±5

^*^Uncertainty is ±2% absolute for Barberton, Santa Catarina, and Santorini and ±4% for Kasos.

^†^The data in this table were taken from ref. 8.

^‡^Error is s.d. rounded up to next full number.
